# The Indiana Veterinary Association

**Published:** 1892-08

**Authors:** J. E. Cloud, W. L. Williams

**Affiliations:** Sec’y, Richmond; President, Lafayette


*The Indiana Veterinary Association.*—After a period of two years of inactivity, a meeting of the Indiana Veterinary Association (Indiana Association of Veterinary Graduates) was convened at the State House, Indianapolis, July 20, 1892, with First Vice-President, C. F. Bell, V. S., of Kokomo, in the chair.

In the absence of the regular secretary, G. G. Ferling, V. S., was appointed secretary *pro tem.*

There were present C. F. Bell, Kokomo; E. F. Diggs, Winchester; M. Y. Schaffer, Greenfield; G. G. Ferling, Richmond; A. D. Galbraith, Greensburg; T. L. Armstrong, Indianapolis; W. L. Williams, Lafayette; J. H. Honan, Delphi; J. E. Cloud, Richmond; Lee Hooven, Richmond; Dr. Klotz, Arcadia; H Y Carnes, Franklin; Prof. L. S. Butler, State College, Miss.

The following new members were elected: W. L. Williams, Purdue University, Lafayette; J. H. Honan, Delphi; J. E. Cloud, Richmond; Lee Hooven, Richmond; Dr. Klotz, Arcadia; H. G. Carnes, Franklin; T. L. Armstrong, Indianapolis, was elected to associate membership.

No election having occurred at the regular time in January, 1892, officers were elected to serve for the remainder of the year as follows: President, W. L. Williams, Lafayette; First Vice-President, C. F. Bell, Kokomo; Second Vice-President, G. G. Ferling, Richmond; Secretary, J. E. Cloud, Richmond; Treasurer, A. D. Galbraith, Greensburg.

The association then listened to a paper by W. L. Williams, entitled "Contagious Pleuro-Pneumonia of the Horse," which was followed by a discussion participated in by Butler, Bell and Honan.

The report of the retiring treasurer, E. F. Diggs, showing a balance on hand of $14 26, was submitted and approved.

The treasurers prior to Dr. Diggs, had apparently been careless in keeping association accounts, and it was found impossible to determine exactly the arrears of dues of each member, and on motion of Dr. Bell, the treasurer elect, was ordered to collect delinquent dues for 1891 and 1892, and receipt therefor. The treasurer was instructed to procure suitable stationery for properly conducting his duties.

On motion of C. F. Bell, it was decided to hold the next meeting January, 1893, at Kokomo.

The president called attention of those present to the meeting of the United States Veterinary Medical Association to be held at Boston, Mass., September 20–22 next, and urged Indiana veterinarians, as far as possible, to attend.

Although the meeting was by no means what could have been desired, the interest and determination displayed by those present, indicated clearly that a united effort is to be made to place this association on a firm footing, and a successful meeting at Kokomo in January is confidently anticipated.

J. E. CLOUD, *Sec'y*,
Richmond.

W. L. WILLIAMS, *President*,
Lafayette.

